# Combined Supplementation of Pre-Exercise Carbohydrate, Alanine, and Proline and Continuous Intake of Green Tea Catechins Effectively Boost Endurance Performance in Mice

**DOI:** 10.3390/nu10070925

**Published:** 2018-07-19

**Authors:** Yoshihiko Minegishi, Atsuko Otsuka, Noriyasu Ota, Koichi Ishii, Akira Shimotoyodome

**Affiliations:** 1 Biological Science Research, Kao Corporation, 2606 Akabane, Ichikai-machi, Haga-gun, Tochigi 321-3497, Japan; minegishi.yoshihiko@kao.com (Y.M.); otsuka.atsuko@kao.com (A.O.); shimotoyodome.akira@kao.com (A.S.); 2 Institute for Innovation, Ajinomoto Co., Inc., Kawasaki, Kanagawa 210-8681, Japan; koichi_ishii@ajinomoto.com

**Keywords:** carbohydrate, fat oxidation, green tea catechins, gluconeogenic amino acids

## Abstract

Continuous intake of green tea catechins (GTC) increases fatty acid utilization as an energy source and improves endurance capacity. Conversely, the single pre-exercise intake of maltodextrin (MD) as a carbohydrate source and the gluconeogenic amino acids alanine (Ala) and proline (Pro) effectively maintain blood glucose levels and increase endurance performance. In this study, we investigated the synergistic combinational effect of these interventions on endurance performance in mice. Male BALB/c mice were fed a 0.5% GTC diet or Control diet for 8 weeks. Maximum running time was measured every 2 weeks. MD (2 g/kg body weight (B.W.)), MD (1 g/kg B.W.) + AlaPro (9:1, 1 g/kg B.W.), and vehicle were orally administrated 60 mins before measurements in each diet group. The GTC + MD + AlaPro group showed significantly higher endurance performance than the Control-Vehicle group at all measurements. Indirect calorimetry analysis during running exercise at 4 weeks in the Control and GTC groups supplemented with pre-exercise MD + AlaPro administration revealed significantly higher fat oxidation in the GTC groups compared to the Control group. The combined increase in fatty acid utilization through continuous GTC intake and pre-exercise MD + AlaPro carbohydrate energy supplementation synergistically improves endurance capacity.

## 1. Introduction

During endurance exercise, skeletal muscle uses glucose and fatty acids as the main energy sources. Glycogen, the storage form of glucose in muscle and liver, is limited, and its depletion causes fatigue [1,2]. Therefore, glycogen sparing is considered an effective and key method to improve endurance capacity and facilitate fatty acid utilization [3,4]. We previously reported that the continuous intake of green tea catechins (GTC) increases fatty acid oxidation in skeletal muscle (the major energy metabolism tissue) and improves endurance capacity by suppressing consumption of muscle glycogen [5,6].

Conversely, effective supplementation of energy sources before and during exercise is also considered a key strategy to improve endurance capacity. For example, glucose intake as a carbohydrate (CHO) source during exercise significantly increases endurance capacity by continuously suppressing decreases in blood glucose [7,8]. Results from several studies have indicated that the single administration of CHO is insufficient to improve endurance performance, while repeated intake of CHO during exercise is very effective to continuously maintain blood glucose levels [9,10]. However, in situations like athletic competition there may be restricted access to supplementation. Thus, it would be beneficial to increase the amount of CHO to a level that sustains blood glucose levels with every intake. Exogenous CHO supplementation, endogenous glycogenolysis, and gluconeogenesis contribute to the maintenance of blood glucose levels during exercise. Nogusa et al. [11] reported that combined ingestion of the gluconeogenic amino acids alanine (Ala) and proline (Pro) with maltodextrin (MD) as a CHO source before exercise effectively maintains blood glucose levels and hepatic glycogen content, and leads to improved endurance performance in mice.

Continuous intake of GTC improves endurance capacity by facilitating metabolic activity to utilize more fatty acids as an energy source, and combined supplementation of Ala and Pro with CHO before exercise immediately contributes to endurance performance by sustainably supplying a CHO energy source during subsequent exercise. These interventions have different mechanisms of action for endurance performance, but they work closely with each other for energy production. Therefore, we hypothesized that the combination of these interventions effectively improves endurance performance. In this study, we investigated this combined effect on endurance performance using treadmill running in mice.

## 2. Materials and Methods

### 2.1. Animals

Male 6-week-old BALB/c mice obtained from Charles River (Kanagawa, Japan) were maintained at 23 ± 2 °C under a 12-h light-dark cycle (lights on from 7:00 to 19:00). All animal experiments were approved by the Animal Care Committee of Kao Corporation. All animal experiments followed this committee's Guidelines for the Care and Use of Laboratory Animals. Before any experiment, mice were fed a laboratory diet (CE-2, CLEA Japan, Tokyo, Japan) to stabilize their metabolism. The macronutrient composition of CE-2 was 4.6% fat, 51.4% carbohydrate, and 24.9% protein.

### 2.2. Materials and Experimental Diets

Polyphenon 70S (Mitsui Norin Co. Ltd., Shizuoka, Japan) used as the GTC mixture contained 80.9% catechins, including epigallocatechin gallate (EGCg, 32.3%), epigallocatechin (EGC, 20%), epicatechin gallate (ECg, 8.8%), epicatechin (EC, 7.9%), gallocatechin gallate (GCg, 3.2%), gallocatechin (GC, 6.1%), catechin (C, 1.8%), and catechin gallate (Cg, 0.8%). Mice were allowed ad libitum access to water and one of two powder diets during experiments. The control diet contained 10% fat (*w/w*), 20% casein, 55.5% potato starch, 8.1% cellulose, 4% minerals, 2.2% vitamins, and 0.2% methionine. The GTC diet consisting of the control diet supplemented with 0.5% GTC. Dietary intake was measured throughout the experimental period by subtracting the weight of the remaining food from the initial weight of the food given on the previous feeding day. Maltodextrin (TK-16, Matsutani Chemical Industry, Hyogo, Japan), DL-Ala (L-Ala: D-Ala was approximately 1:1), and L-Pro were provided from Ajinomoto Co., Inc. (Tokyo, Japan). MD, DL-Ala, and L-Pro were dissolved in saline as a vehicle immediately before use.

### 2.3. Experimental Design

We performed two experiments in this study. In Experiment 1, we evaluated the change over time in running endurance capacity with interventions. In Experiment 2, we focused on energy metabolic status during running exercise.

#### 2.3.1. Experiment 1

Male 6-week-old BALB/c mice were allowed ad libitum access to water and the CE-2 diet for acclimation. At 7 weeks of age, mice were assigned to the 3-day treadmill running training (details of treadmill conditions are described in Section 2.4.). At 8 weeks of age, initial running endurance capacity was measured for 104 of the 120 mice that had adapted to the running exercise without problems. To minimize variations in endurance performance, 56 of 104 mice were selected centering on the overall average running time and then divided into seven experimental groups (*n* = 8) with similar average running times and body weight (B.W.) (Table 1).

During the experimental period, mice were fed the Control or 0.5% GTC diet and subjected to a 30 mins treadmill running exercise 4 days a week, except for the Non-Ex group. After 2, 4, 6, and 8 weeks from the start of the experiment, endurance capacity was evaluated for each pre-exercise administration using the same methods as the initial measurement. Mice were orally administrated 10 mL/kg B.W. vehicle, MD (2.0 g/kg B.W.), or MD with supplemented AlaPro (1.0 g/kg MD + 0.9 g/kg DL-Ala + 0.1 g/kg L-Pro) solution 60 mins before evaluation of endurance capacity. Two days after final endurance measurements at week 8, mice were anesthetized with isoflurane (Abbott Japan, Tokyo, Japan). Subsequently, blood and tissues, such as liver and skeletal muscles (gastrocnemius, soleus, quadriceps, and tibialis anterior muscles), were collected and weighed. The tissues were immediately frozen in liquid nitrogen and stored at −80 °C.

#### 2.3.2. Experiment 2

According to the same procedure in Experiment 1, 16 of 32 male 8-week-old BALB/c mice were selected and divided into the Control and 0.5% GTC diet group, with similar average running times (145.5 ± 7.6 and 145.2 ± 7.6, respectively) and B.W. Both groups were fed each experimental diet and exercised by treadmill running 4 days a week. Energy metabolism during running was determined by indirect calorimetry analysis (described in Section 2.5) at week 4. At week 6, both mouse groups were fasted for 2 h and then orally administrated MD supplemented with AlaPro (1.0 g/kg MD + 0.9 g/kg DL-Ala + 0.1 g/kg L-Pro) 60 mins before exercise. Blood and tissue samples were collected from anesthetized mice immediately after 60 mins of running at 20 m/min.

### 2.4. Running Exercise and Evaluation of Endurance Performance

A 10-lane motorized rodent treadmill (MK-680; Muromachi Kikai, Tokyo, Japan) with an 8° incline was used for the running exercise and to evaluate endurance capacity. The initial 3-day treadmill training was conducted according to the following program: day 1, 10 m/min for 10 mins and 15 m/min for 20 mins; day 2, 10 m/min for 10 mins, 15 m/min for 10 mins, and 20 m/min for 10 mins; and day 3, 15 m/min for 10 mins, 20 m/min for 10 mins, and 25 m/min for 10 min. Evaluation of endurance capacity was according to following program: 10 m/min for 6 mins, 12 m/min for 2 mins, 14 m/min for 2 mins, 16 m/min for 2 mins, 18 m/min for 2 mins, 20 m/min for 2 mins, 22 m/min for 2 mins, 24 m/min for 2 mins, 26 m/min for 2 mins, and 28 m/min until exhaustion. The daily running exercise program during the experimental period was 15 m/min for 10 mins and 20 m/min for 20 mins 4 days a week.

### 2.5. Indirect Calorimetry Analysis under Exercise Conditions

In Experiment 2, energy metabolism analyses under running exercise conditions were performed by using an ARCO-2000 magnetic-type mass spectrometric calorimeter (ARCO System, Chiba, Japan) with a four-lane airtight rodent treadmill with 5° incline setting (Modular Treadmill System; Columbus Instruments, Columbus, OH, USA), with one mouse per lane. Mice were fasted 120 mins before measurements, and MD supplemented with AlaPro (1.0 g/kg MD + 0.9 g/kg DL-Ala + 0.1 g/kg L-Pro) were orally provided to all mice 60 mins before measurements. Mice were placed in a treadmill chamber for 30 mins before measurements to acclimate to the surroundings. Mice ran with an initial speed of 10 m/min for 5 mins, to adapt to running gradually on the treadmill. The running speed was then changed to 15 m/min for 5 mins and subsequently to 20 m/min for 50 mins. Data were collected every 75 secs throughout the 60-min running period. The respiratory exchange ratio (RER) was calculated from the measured values of oxygen consumption (VO_2_) and carbon dioxide exhalation (VCO_2_), and fat oxidation and carbohydrate oxidation were calculated using the following equations [12]:

RER = VCO_2_/VO_2_
Fat oxidation (mg/min/kg B.W.) = 1.695 × (1 − 1.701/1.695 × RER) × VO_2_
Carbohydrate oxidation (mg/min/kg B.W.) = (4.585 × RER − 3.226) × VO_2_

For data comparison between the MD + AlaPro and GTC + MD + AlaPro group, the measurements at the 20 m/min for 50 mins portion of the run were selected for analysis to avoid the effect of a rapid change once running was initiated.

### 2.6. Liver and Muscle Glycogen Contents

Liver and gastrocnemius muscle glycogen contents after the 60-min running exercise in Experiment 2 were measured as described previously [6]. In brief, 10 mg of liver and 50 mg of gastrocnemius muscle were digested in 300 L of 30% KOH for 30 mins in a boiling water bath. After 50 L of saturated sodium sulfate was added, the glycogen was precipitated by adding 500 L of 95% ethanol and centrifuged at 1600× *g*. The supernatant was decanted and the remaining ethanol was vaporized in an incubator at 80 °C for 15 mins. The pellet was dissolved in 200 L of H_2_O and reprecipitated with 250 L of 95% ethanol. The supernatant was decanted after centrifugation at 1600× g, and the remaining alcohol was vaporized. Purified glycogen was hydrolyzed in 600 L of 0.6 N HCl at 100 °C for 3 h. Glucose levels were determined with the Glucose CII test kit (Wako Pure Chemical Industries, Wako, Japan) and converted to the glycogen concentration.

### 2.7. Statistical Analyses

All values are presented as mean ± standard error of the mean (SE). Statistical analysis was conducted using ANOVA followed by the Dunnett’s test with the Control-Vehicle group in Experiment 1. In Experiment 2, the comparison of the time course changes in the indirect calorimetry analysis between Control and GTC diet group running at 20 m/min was performed using two-way repeated measures ANOVA. This analysis was performed to determine the main effects corresponding to group and time, as well as the interaction between the two. The unpaired *t*-test was used to compare between two groups. A *P*-value < 0.05 was considered statistically significant. Data were organized and analyzed using the Microsoft Excel 2010 (Microsoft Corp., Redmond, WA, USA) and GraphPad Prism 6 (GraphPad software Inc., La Jolla, CA, USA).

## 3. Results

### 3.1. Effect on Endurance Performance

**Figure 1:**
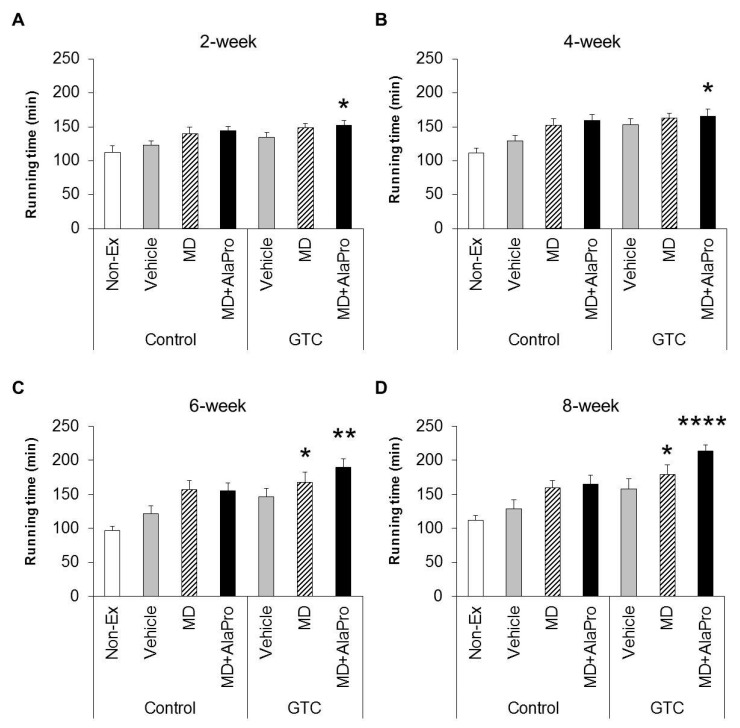
Running time to exhaustion as measured to evaluate endurance capacity at the (**A**) 2, (**B**) 4, (**C**) 6, and (**D**) 8-week test period. Values are means ± SE (*n* = 8, Non-Ex and GTC + MD at 8-week: *n* = 7). * *P* < 0.05, ** *P* < 0.01, **** *P* < 0.0001 vs. Control-Vehicle group (Dunnett’s test).

We evaluated the combinational effects of pre-exercise CHO, Ala, and Pro supplementation and continuous GTC ingestion on improvements in endurance performance at 2, 4, 6, and 8-week interventions. At the 2-week measurement, in the Control diet group, MD and MD + AlaPro increased running time by 13.6% and 17.0%, respectively, compared to Vehicle. However, these differences were not statistically significant. In the GTC diet group, Vehicle and MD increased running time by 9% and 20.6%, respectively; these differences were also not significant. In contrast, the GTC + MD + AlaPro group significantly increased running time by 23.9% compared to that in the Control-Vehicle group (Figure 1A).

At the 4-week measurement, MD + AlaPro, and not MD alone, in the Control diet group tended to increase running time by 23% (*P* = 0.097), compared to that by the Vehicle (Figure 1B). In the GTC diet group, Vehicle displayed 18.3% increased endurance performance, which was twice that of the 2-week measurement, indicating that the GTC effect began to appear strongly at week 4; however, the effect was still not significant. GTC + MD tended to increase running time by 25.6% (*P* = 0.052) and GTC + MD + AlaPro significantly increased running time by 28.3% compared to that of the Control-Vehicle group. At the 6-week measurement, MD and MD + AlaPro in the GTC diet group significantly increased the running time significantly by 38.4% and 55.7%, respectively, compared to the Control-Vehicle group (Figure 1C). After the 6-week measurement, two mice (one each from the Non-Ex and GTC + MD groups) were excluded from the subsequent experiment due to injury. At the 8-week measurement, MD and MD + AlaPro in the Control diet group and Vehicle in the GTC diet group showed a 24.4%, 28.5%, and 22.7% increase in running time, respectively, compared to that in the Vehicle group; however, these differences were not significant. The GTC + MD group displayed a significant 40% increase in running time compared to that by the Control-Vehicle group. In addition, the GTC + MD + AlaPro group showed a significant 66.4% increase, the highest rate of increase in this study, compared to the Control-Vehicle group (Figure 1D).

**Figure 2:**
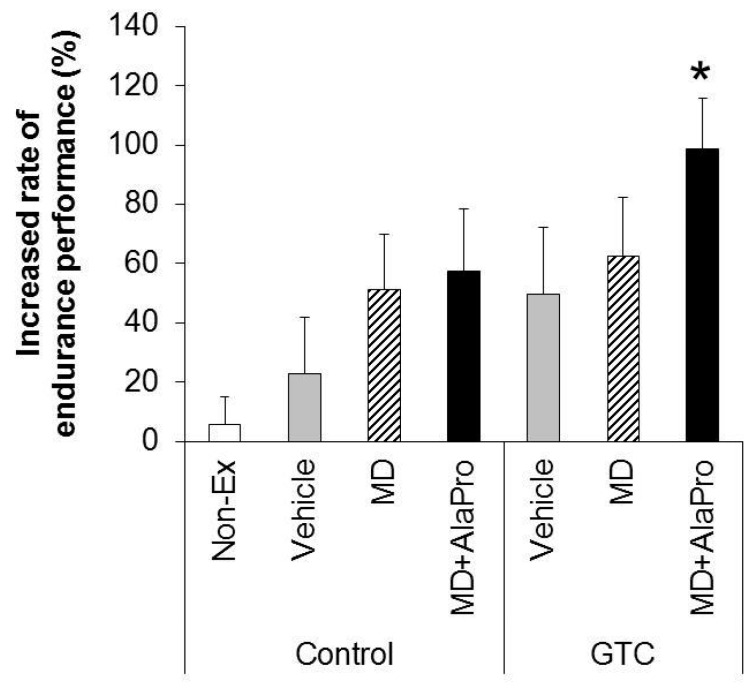
Increased rate of endurance performance after 8-week intervention. Values are means ± SE (*n* = 8, Non-Ex and GTC + MD: *n* = 7). * *P* < 0.05 vs. Control-Vehicle group (Dunnett’s test).

The average rates of increase of endurance performance from the initial time to the 8-week intervention in each group are presented in Figure 2. The Control-Vehicle group displayed an increase of approximately 22.9% from initial running time. However, this rate of increase was not significantly different from that of the Non-Ex group, suggesting that the 8-week running training had relatively less impact on endurance performance. GTC + MD + AlaPro produced an increase of 98.8% from initial performance, which was significantly higher than that of the Control-Vehicle group.

### 3.2. Body and Tissue Weights

Body and tissue weights at the end of this study are shown in Table 2. There were no significant differences in body weight, liver weight, and lower limb muscle weight between the intervention groups. Average food intakes of each group during the test periods were as follows: Non-Ex (3.10 g/day/mouse), Control-Vehicle (3.00 g/day/mouse), MD (3.13 g/day/mouse), MD + AlaPro (3.15 g/day/mouse), GTC (3.09 g/day/mouse), GTC + MD (3.10 g/day/mouse), and GTC + MD + AlaPro (3.12 g/day/mouse).

### 3.3. Effect on Energy Metabolism during Running Exercise

**Figure 3:**
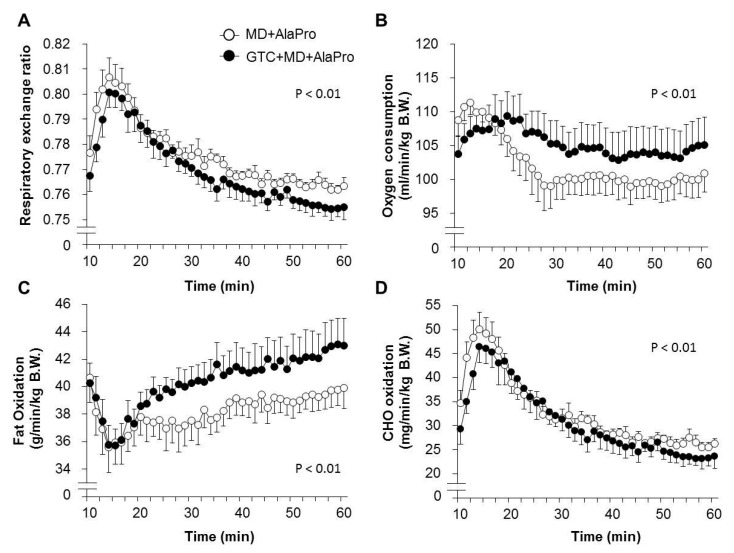
Energy metabolism during running exercise. (**A**) RER, (**B**) oxygen consumption, (**C**) fat oxidation, and (**D**) CHO oxidation were measured by indirect calorimetry during a 60-min treadmill running exercise as described in the Methods section. Mean values for each time points are plotted. Values are means ± SE (*n* = 8). *P* values reflect between-group differences assessed by two-way repeated-measures ANOVA during the 50-min running period at 20 m/min.

To elucidate the combinational effect of continuous GTC intake and pre-exercise MD + AlaPro on energy metabolism during running exercise, we performed indirect calorimetry analysis in both control and GTC diet fed groups supplemented with pre-exercise MD + AlaPro administration in Experiment 2. Two-way repeated measures ANOVA indicated significant differences in groups and time for RER, oxygen consumption, fat oxidation, and CHO oxidation during the 50-min running at a speed of 20 m/min, with no significant interaction between these parameters. GTC + MD + AlaPro showed lower RER (Figure 3A) and higher oxygen consumption (Figure 3B) than the results using MD + AlaPro alone. GTC + MD + AlaPro showed higher fat oxidation (Figure 3C) and lower CHO oxidation (Figure 3D) than those of MD + AlaPro alone.

### 3.4. Effect on Glycogen Storage in Liver and Skeletal Muscle

**Figure 4:**
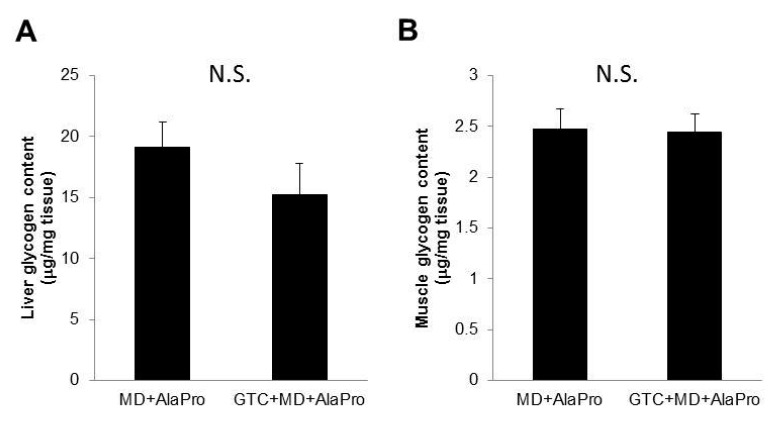
Glycogen storage in (**A**) liver and (**B)** gastrocnemius muscle after running exercise. Both control and GTC group mice were fed MD + AlaPro 60 mins before running and ran on the treadmill for 60 mins at 20 m/min. Subsequently, the liver and muscles were collected. Values are means ± SE (*n* = 8). N.S., not significant.

In Experiment 2, we measured glycogen storage in liver and gastrocnemius muscle after the 60-min running exercise. Under the same conditions in which MD + AlaPro was administered 60 mins before running, there were no significant differences between control and GTC fed mice in both liver and gastrocnemius muscle glycogen content (Figure 4).

## 4. Discussion and Conclusions

Our main findings in this study are as follows: (1) pre-exercise single intake of CHO with Ala and Pro (MD + AlaPro) increased the endurance improving effect of continuous GTC intake more effectively than CHO alone and (2) the increase of sustained fat utilization for energy production during running exercise potentially contributed to this combinational effect.

MD + AlaPro increased the effectiveness of improved endurance performance by dietary GTC intake and succeeded in eliciting a significant effect at early 2- and 4-week GTC intervention. The increased rate of endurance performance from the initial time to week 8 of intervention was significantly higher only in the GTC + MD + AlaPro group compared to the Control-Vehicle group. These results suggest that pre-exercise MD + AlaPro intake is quite effective for enhancing the endurance improving effect by continuous GTC intake. In the Control diet group, both MD + AlaPro and MD alone tended to increase endurance performance compared to the Vehicle group. The difference was not significant. In contrast, MD + AlaPro was more effective in endurance improvement than MD alone in a previous study [11]. We think that the difference in treadmill conditions while evaluating endurance performance could have had a profound effect on this difference between the two studies. The treadmill speed during endurance measurement in the previous study (36 m/min) [11] was much faster than the speed of 28 m/min used in our study. Since CHO metabolism has a greater influence on energy metabolism at high exercise intensity, the difference between MD and MD + AlaPro was clearly observed in the previous study, whereas our current measurement conditions might be more likely to be affected by lipid metabolism, which is the main mechanism of GTC, than by CHO metabolism. In any case, MD + AlaPro administration was able to consistently increase endurance capacity by only a single intake at any measurement timing. Conversely, GTC showed gradually increased effectiveness in endurance with continuous intake but was less effective in the early stage of intervention. Endurance was synergistically improved by MD + AlaPro in proportion to continuous GTC intake, suggesting that combining these interventions could be beneficial as a comprehensive control method for energy metabolism during exercise.

Fatty acids have higher energy production capacity and larger storage amounts in the body compared to CHO, therefore, enhancing lipid utilization can be an effective strategy for improving endurance [4]. Fatty acids are metabolized to acetyl-CoA in the mitochondria and converted into energy by entering the tricarboxylic acid (TCA) cycle. Although acetyl-CoA is also supplied from CHO through glycolysis, an increased supply of acetyl-CoA from fatty acid could lead to the suppression of carbohydrate utilization and glycogen sparing. Previous studies revealed that continuous GTC intake significantly decreased RER during exercise, accompanied by high fatty acid-oxidation in skeletal muscle [5,6]. In this study, we confirmed higher fat oxidation in the GTC diet group during running exercise than the Control diet group under the same pre-exercise conditions of MD + AlaPro administration. This upregulated fatty acid utilization by continuous GTC intake and the sustained blood glucose maintenance by pre-exercise MD + AlaPro intake might contribute to efficient energy production via acetyl-CoA from fatty acids, resulting in improved endurance capacity.

During prolonged exercise, increases in TCA cycle intermediates are also needed to sustain sufficient aerobic energy production [13,14]. To produce ATP through the TCA cycle reaction, oxaloacetate needs to combine with acetyl-CoA and convert to citrate. With increased ATP consumption in the muscle during exercise, increased oxaloacetate induces citrate synthase activation, resulting in ATP production in the TCA cycle and fatty acid oxidation. Endurance exercise training induces fatty acid utilization through the coordinated activation of the mitochondrial fatty acid oxidation enzyme complex and citrate synthase in the skeletal muscle [15,16], indicating the importance of the TCA cycle on endurance performance. CHO supplementation before and during exercise increases TCA intermediates in the muscle and contributes to improved endurance performance [17,18]. In addition to the role as CHO supplier, the gluconeogenic amino acids, Ala and Pro, are converted to the respective TCA intermediates of oxaloacetate and alpha-ketoglutalate. In the condition that GTC intake increases acetyl-CoA supply through fatty acid oxidation, increased oxaloacetate supply may contribute to the promotion of the TCA cycle reaction. Since pyruvate from glycolysis and Ala are the sources of oxaloacetate, combinations to increase oxaloacetate and acetyl-CoA from fatty acid oxidation through GTC intake may synergistically contribute to TCA cycle facilitation and ATP production. To clarify the precise mechanism in the combinational effect of GTC + MD + AlaPro, further studies including determination of time-course changes in blood components related to energy metabolism, such as glucose, lactate, fatty acid, and amino acids, and TCA intermediates in muscles during exercise, are needed.

We hypothesized that GTC + MD + AlaPro administration would result in glycogen sparing and contribute to increased endurance performance. However, we could not find a difference in liver and muscle glycogen storage after exercise between the MD + AlaPro and GTC + MD + AlaPro groups. To observe the effect on glycogen sparing, we loaded a 60-min running exercise at the 20 m/min speed for glycogen consumption. This is based on the following two reasons. First, it was achievable for all mice according to the endurance measurements in Experiment 1. Second, an increase in fat oxidation was stably observed during the running exercise by indirect calorimetry analysis in Experiment 2. In fact, the minimum running time for mice fed the MD + AlaPro condition was over 130 mins in this study. Therefore, the 60-min running load might not have been enough to observe differences in glycogen consumption. To confirm the precise effect on glycogen sparing, we need to determine appropriate test conditions for glycogen consumption in another study.

In conclusion, the combination of increased fatty acid utilization by continuous GTC intake and effective pre-exercise CHO energy supplementation by MD + AlaPro synergistically improves endurance capacity. These observations indicate that combining nutritional interventions based on different mechanisms of action in energy metabolism may be an effective strategy to improve endurance performance.

## Figures and Tables

**Table 1:** Experimental groups.

Group	Diet	Pre-Exercise Administration	Average Initial Running Time (min)
Non-exercise control (Non-Ex)	Control	Vehicle	111.6 ± 7.4
Control-Vehicle	Control	Vehicle	111.7 ± 7.3
MD	Control	MD (2 g/kg B.W.)	111.5 ± 7.3
MD + AlaPro	Control	MD (1 g/kg B.W.) + AlaPro (1 g/kg B.W.)	111.5 ± 7.4
GTC-Vehicle	0.5% GTC	Vehicle	111.8 ± 7.2
GTC + MD	0.5% GTC	MD (2 g/kg B.W.)	111.5 ± 8.1
GTC + MD + AlaPro	0.5% GTC	MD (1 g/kg B.W.) + AlaPro (1 g/kg B.W.)	111.5 ± 8.1

Values are means ± SE (*n* = 8).

**Table 2:** Body and tissue weights.

Group	Body Weight (g)	Liver (g)	Soleus (mg)	Gastrocnemius (mg)	Quadriceps (mg)
Non-Ex	32.1 ± 0.4	1.28 ± 0.04	23.0 ± 0.4	299.2 ± 6.3	451.4 ± 13.4
Control-Vehicle	31.0 ± 0.3	1.25 ± 0.02	22.5 ± 0.7	287.8 ± 5.1	424.4 ± 21.7
MD	31.9 ± 0.7	1.24 ± 0.03	23.2 ± 0.9	297.6 ± 5.9	436.7 ± 10.3
MD + AlaPro	31.6 ± 0.7	1.23 ± 0.03	23.1 ± 0.5	285.8 ± 6.6	464.0 ± 15.0
GTC-Vehicle	31.1 ± 0.4	1.22 ± 0.04	22.2 ± 0.6	295.1 ± 4.2	439.4 ± 15.7
GTC + MD	32.1 ± 0.8	1.30 ± 0.02	25.0 ± 1.8	295.1 ± 3.8	459.0 ± 11.3
GTC + MD + AlaPro	32.6 ± 0.6	1.31 ± 0.02	22.9 ± 0.8	298.7 ± 3.7	452.7 ± 9.0

Values are means ± SE (*n* = 8, Non-Ex and GTC + MD: *n* = 7).

## References

[1] Holloszy J.O., Kohrt W.M., Hansen P.A. (1998). The regulation of carbohydrate and fat metabolism during and after exercise. Front. Biosci..

[2] Ivy J.L. (1999). Role of carbohydrate in physical activity. Clin. Sports Med..

[3] Hawley J.A., Brouns F., Jeukendrup A. (1998). Strategies to enhance fat utilisation during exercise. Sports Med..

[4] Jeukendrup A.E., Saris W.H., Wagenmakers A.J. (1998). Fat metabolism during exercise: A review—Part III: Effects of nutritional interventions. Int. J. Sports Med..

[5] Murase T., Haramizu S., Shimotoyodome A., Nagasawa A., Tokimitsu I. (2005). Green tea extract improves endurance capacity and increases muscle lipid oxidation in mice. Am. J. Physiol. Regul. Integr. Comp. Physiol..

[6] Murase T., Haramizu S., Shimotoyodome A., Tokimitsu I., Hase T. (2006). Green tea extract improves running endurance in mice by stimulating lipid utilization during exercise. Am. J. Physiol. Regul. Integr. Comp. Physiol..

[7] Ivy J.L., Miller W., Dover V., Goodyear L.G., Sherman W.M., Farrell S., Williams H. (1983). Endurance improved by ingestion of a glucose polymer supplement. Med. Sci. Sports Exerc..

[8] Coyle E.F., Coggan A.R., Hemmert M.K., Ivy J.L. (1986). Muscle glycogen utilization during prolonged strenuous exercise when fed carbohydrate. J. Appl. Physiol..

[9] Febbraio M.A., Chiu A., Angus D.J., Arkinstall M.J., Hawley J.A. (2000). Effects of carbohydrate ingestion before and during exercise on glucose kinetics and performance. J. Appl. Physiol..

[10] Sparks M.J., Selig S.S., Febbraio M.A. (1998). Pre-exercise carbohydrate ingestion: Effect of the glycemic index on endurance exercise performance. Med. Sci. Sports Exerc..

[11] Nogusa Y., Mizugaki A., Hirabayashi-Osada Y., Furuta C., Ohyama K., Suzuki K., Kobayashi H. (2014). Combined supplementation of carbohydrate, alanine, and proline is effective in maintaining blood glucose and increasing endurance performance during long-term exercise in mice. J. Nutr. Sci. Vitaminol..

[12] Péronnet F., Massicotte D. (1991). Table of nonprotein respiratory quotient: an update. Can. J. Sport Sci..

[13] Sahlin K., Katz A., Broberg S. (1990). Tricarboxylic acid cycle intermediates in human muscle during prolonged exercise. Am. J. Physiol..

[14] Gibala M.J., Tarnopolsky M.A., Graham T.E. (1997). Tricarboxylic acid cycle intermediates in human muscle at rest and during prolonged cycling. Am. J. Physiol..

[15] Carter S.L., Rennie C.D., Hamilton S.J., Tarnopolsky (2001). Changes in skeletal muscle in males and females following endurance training. Can. J. Physiol. Pharmacol..

[16] Powers S.K., Lawler J., Criswell D., Lieu F.K., Martin D. (1992). Aging and respiratory muscle metabolic plasticity: Effects of endurance training. J. Appl. Physiol..

[17] Spencer M.K., Yan Z., Katz A. (1991). Carbohydrate supplementation attenuates IMP accumulation in human muscle during prolonged exercise. Am. J. Physiol..

[18] Spencer M.K., Yan Z., Katz A. (1992). Effect of low glycogen on carbohydrate and energy metabolism in human muscle during exercise. Am. J. Physiol..

